# Efeitos das emendas parlamentares no financiamento municipal da atenção primária à saúde do Sistema Único de Saúde 

**DOI:** 10.1590/0102-311XPT007323

**Published:** 2024-04-22

**Authors:** Karla Giovana Bavaresco Ulinski, Brígida Gimenez Carvalho, Fabiola Sulpino Vieira, Renne Rodrigues, Luciana Dias de Lima

**Affiliations:** 1 Universidade Estadual de Londrina, Londrina, Brasil.; 2 Instituto de Pesquisa Econômica Aplicada, Brasília, Brasil.; 3 Escola Nacional de Saúde Pública Sergio Arouca, Fundação Oswaldo Cruz, Rio de Janeiro, Brasil.

**Keywords:** Financiamento da Assistência à Saúde, Atenção Primária à Saúde, Gastos em Saúde, Alocação de Recursos, Sistema Único de Saúde, Healthcare Financing, Primary Health Care, Health Expenditures, Resource Allocation, Unified Health System, Financiación de la Atención de la Salud, Atención Primaria de Salud, Gastos en Salud, Asignación de Recursos, Sistema Único de Salud

## Abstract

O objetivo deste artigo é analisar os efeitos da ampliação do repasse federal de emendas parlamentares no financiamento municipal da atenção primária à saúde (APS) do Sistema Único de Saúde (SUS), no período de 2015 a 2020. Foi realizado estudo longitudinal com dados secundários de transferências por emendas parlamentares do Ministério da Saúde e de despesas com recursos próprios dos municípios, aplicadas em ações e serviços públicos de saúde e na APS. O efeito do repasse de emendas parlamentares no financiamento municipal foi verificado de forma estratificada por porte populacional dos municípios, por meio de modelos de equações de estimativas generalizadas. O repasse de emendas parlamentares para a APS apresentou grande discrepância de valores *per capita* entre os municípios de diferentes portes populacionais. Observou-se inexistência de correlação com a despesa municipal em ações e serviços públicos de saúde nos municípios com mais de 10 mil habitantes e associação inversa com a despesa em APS (p < 0,050) em todos os grupos. Conclui-se que o aumento do repasse de emendas parlamentares pelo Ministério da Saúde favoreceu a redução da alocação de receitas municipais com APS, que podem ter sido direcionados para outras finalidades de gasto no SUS. Tais mudanças parecem refletir prioridades estabelecidas para a despesa orçamentária dos municípios, que repercutem sobre as condições locais para a garantia da estabilidade do financiamento da APS no Brasil.

## Abstract (en)

This study aims to analyze the effects of the expansion of the federal transfer of parliamentary amendments for municipal financing of primary health care (PHC) in the Brazilian Unified National Health System (SUS), from 2015 to 2020. A longitudinal study was conducted using secondary data on transfers of parliamentary amendments from the Brazilian Ministry of Health and expenditure of municipalities’ own resources on public health actions and services and PHC. The effect of the transfer of parliamentary amendments on municipal financing was verified in a stratified way by population size of the municipalities, using generalized estimating equation models. The transfer of parliamentary amendments for PHC showed a large discrepancy in per capita values among municipalities of different population sizes. No correlation with municipal spending on public health actions and services was observed in municipalities with more than 10,000 inhabitants, and the association with spending on PHC (p < 0.050) was inverse in all municipalities. Therefore, the increase in the transfer of parliamentary amendments by the Brazilian Ministry of Health favored a reduction in the allocation of municipal revenues to PHC, which may have been directed to other spending purposes in the SUS. These changes seem to represent priorities established for municipal budget expenditure, which have repercussions on local conditions for guaranteeing stable funding for PHC in Brazil.

## Abstract (es)

El artículo tiene como objetivo analizar los efectos de la ampliación de la transferencia de recursos federal de enmiendas parlamentarias sobre el financiamiento municipal de la atención primaria de salud (APS) en el Sistema Único de Salud brasileño (SUS), en el período del 2015 al 2020. Se realizó un estudio longitudinal con datos secundarios de transferencias de recursos por enmiendas parlamentarias del Ministerio de Salud y de gastos con recursos propios de los municipios, aplicados a acciones y servicios públicos de salud y a la APS. El efecto de la transferencia de recursos de enmiendas parlamentarias sobre el financiamiento municipal se verificó de forma estratificada por tamaño de población de los municipios, utilizando modelos de ecuaciones de estimaciones generalizadas. La transferencia de recursos de enmiendas parlamentarias para la APS mostró una gran discrepancia en los valores per cápita entre municipios de diferente tamaño poblacional. No hubo correlación con el gasto municipal en acciones y servicios públicos de salud en aquellos con más de 10.000 habitantes y asociación inversa con el gasto en APS (p < 0,050) en todos los grupos de municipios. Se concluye que el aumento en la transferencia de recursos de enmiendas parlamentarias por parte del Ministerio de Salud favoreció la reducción de la asignación de ingresos municipales a la APS, que pueden haber sido dirigidos a otros fines de gasto en el SUS. Tales cambios parecen reflejar prioridades establecidas para el gasto presupuestario municipal, que repercuten en las condiciones locales para garantizar la estabilidad del financiamiento de la APS en Brasil.

## Introdução

O financiamento insuficiente do Sistema Único de Saúde (SUS) é apontado como um dos maiores obstáculos à garantia do direito à saúde no Brasil nos termos da *Constituição Federal* de 1988 ^1^^,^^2^. Os esforços das entidades e dos movimentos sociais em defesa do financiamento adequado da saúde, junto ao Congresso Nacional, conquistaram a vinculação de recursos, determinando que a União, os estados, o Distrito Federal e os municípios realizem gastos mínimos em saúde ^3^. Durante os anos 2000, a vigência da vinculação orçamentária contribuiu para a estabilização da participação do gasto federal no produto interno bruto (PIB) e para o crescimento do aporte de recursos dos estados e, especialmente, dos municípios no financiamento do SUS ^4^^,^^5^^,^^6^. Contudo, a vinculação não foi suficiente para assegurar o financiamento do acesso aos bens e serviços de saúde de forma universal, igualitária e integral em todo o país ^7^.

Nos últimos anos, as limitações para o financiamento do SUS se intensificaram em decorrência das crises econômica e política do período de 2014 a 2016, com o fortalecimento da agenda neoliberal no Governo Federal, resultando na implementação de políticas drásticas de austeridade fiscal a partir de 2016 ^8^^,^^9^. Nesse contexto, a insatisfação do Congresso Nacional quanto à execução orçamentária e financeira de emendas parlamentares pela União culminou na aprovação do orçamento impositivo. Inicialmente por força da *Lei de Diretrizes Orçamentárias* de 2014 e, nos anos subsequentes, por determinação da *Emenda Constitucional nº 86/2015* (EC 86), a execução das emendas individuais passou a ser obrigatória pelo Executivo Federal, salvo os casos com impedimentos de ordem técnica ^10^. Posteriormente, a *Emenda Constitucional nº 100/2019* (EC 100) estendeu essa compulsoriedade para as emendas de bancada estaduais ^11^.

A EC 86 estabeleceu que metade do limite das emendas individuais deve ser alocado ao financiamento de ações e serviços públicos de saúde, o que corresponde a 0,6% da receita corrente líquida da União. Com isso, o Ministério da Saúde passou a autorizar a execução de emendas parlamentares para o custeio da atenção primária e de média e alta complexidade, por meio do incremento do Piso da Atenção Básica (PAB) e do incremento do teto de média e alta complexidade, respectivamente ^10^. Além disso, no ano de 2022, com a aprovação da *Emenda Constitucional nº 126/2022* (EC 126), uma nova alteração do limite para a execução de emendas individuais ampliou a alocação desses recursos para a saúde, passando a ser de 1% sobre a receita corrente líquida ^12^.

Tais medidas aumentaram ainda mais a complexidade do financiamento do SUS, tendo em vista que a atuação parlamentar passou a ter uma relevância maior na destinação dos recursos federais, em meio à severa restrição orçamentária imposta ao Ministério da Saúde com o congelamento de sua aplicação mínima em ações e serviços públicos de saúde pela *Emenda Constitucional nº 95/2016* (EC 95) (teto de gastos) ^13^. Ademais, além do crescimento da execução de despesas por emendas parlamentares impositivas, o Ministério da Saúde expandiu a execução de despesas por emendas parlamentares de relator, que não são obrigatórias, ampliando significativamente o espaço das emendas nos recursos federais alocados para a saúde pública ^10^.

No Brasil, a participação das emendas parlamentares no orçamento federal é tratada, na literatura científica, com abordagens e enfoques distintos. Parte dos trabalhos concentram-se nos anos 2000 e estão direcionados para a compreensão da dinâmica das relações entre Executivo e Legislativo e suas repercussões para as políticas públicas. Com esse propósito, alguns autores enfatizam as emendas parlamentares como instrumento clientelista utilizado pelos parlamentares com fins eleitorais, o que contribui para a iniquidade na alocação de recursos públicos ^14^^,^^15^. Entretanto, outros estudos procuram refutar esse argumento. Por um lado, destacam a força do Executivo no direcionamento das emendas parlamentares e a atuação disciplinada dos legisladores para apoiar e fortalecer iniciativas coletivas e programas implementados pelo governo ^16^^,^^17^^,^^18^. Por outro, tem importância como meio para conciliar interesses nacionais e locais, possibilitando diminuir a iniquidade decorrente das regras da partilha fiscal entre os diferentes entes da Federação ^19^.

Embora pesquisas voltadas especificamente para a análise da atuação do Legislativo no financiamento da saúde tenham sido desenvolvidas ainda no início de 2010 ^20^, elas se ampliaram a partir de 2015, no contexto do orçamento impositivo. Isso porque a obrigatoriedade da execução de emendas parlamentares pelo Executivo Federal tende a interferir no processo de coordenação da implantação do SUS, fortemente ancorado nos mecanismos de transferência de recursos programáticos do Ministério da Saúde para os municípios ^21^. Essa forma de repasse, instituída e ampliada a partir da segunda metade dos anos 1990, favoreceu a adoção de parâmetros nacionais de políticas específicas e contribuiu, embora com limitações, para a redução das desigualdades regionais no país ^13^.

O aumento da participação das emendas parlamentares no financiamento da saúde suscitou preocupações com o desinvestimento em políticas estruturantes do SUS ^22^^,^^23^, com a baixa qualidade, a adequação do gasto público às necessidades locais ^24^^,^^25^ e as distorções da alocação de recursos federais sob a ótica da equidade ^26^.

Visando contribuir com esse debate, este artigo busca analisar os efeitos da ampliação do repasse federal de emendas parlamentares no financiamento municipal da atenção primária do SUS, no período de 2015 a 2020. A justificativa para esse recorte está ancorada na relevância da participação do gasto municipal para o financiamento de ações e serviços públicos de saúde e da atenção primária à saúde (APS).

De 2004 a 2017, o percentual médio de aplicação do conjunto de municípios em todos os estados da Federação superou os 15% de recursos próprios (somatório do gasto financiado por receitas de impostos diretamente arrecadadas e por transferências constitucionais e legais), que devem ser alocados ao SUS ^3^. Destaca-se que a APS é uma das principais áreas de gasto municipal em saúde ^27^. A regulação e a destinação programática de recursos federais para a APS ^28^, associadas à pressão da demanda por ampliação da cobertura de serviços ^29^, contribuíram para a descentralização e para o maior esforço de alocação de receitas pelos municípios, para além do mínimo obrigatório para a saúde.

Neste estudo, procuramos investigar se a ampliação dos repasses federais por emendas parlamentares favoreceu a redução da alocação de recursos próprios pelos municípios para o financiamento de ações e serviços públicos de saúde e APS, além de discutir o significado e as consequências das mudanças orçamentárias observadas para o SUS.

## Método

Trata-se de um estudo longitudinal e quantitativo, desenvolvido no âmbito de um projeto multicêntrico, aprovado pelo Comitê de Ética da Escola Nacional de Saúde Pública Sergio Arouca (ENSP) da Fundação Oswaldo Cruz (Fiocruz) (CAAE: 30675420.6.0000.5240).

Dados secundários de execução de despesas em saúde do Ministério da Saúde e dos municípios foram utilizados na investigação da associação entre as transferências de recursos federais por emendas parlamentares e a despesa municipal em ações e serviços públicos de saúde e APS.

O trabalho se baseia na teoria moderna do orçamento público, que o define como instrumento de gestão de natureza diversa: expressa escolha de agentes políticos; envolve alocação de recursos financeiros; constitui um plano de aplicação; e traduz uma lei ^30^^,^^31^ . Por meio da análise do orçamento, é possível verificar prioridades governamentais e políticas e suas consequências para o financiamento e a gestão de políticas públicas.

### Características do estudo e variáveis

Foram analisados dados de transferência federal por emendas parlamentares e de despesa com ações e serviços públicos de saúde e APS dos 5.568 municípios brasileiros no período de 2015 a 2020, incluídos na base de dados construída para o projeto. Fernando de Noronha e Brasília não foram incluídos porque, embora constem da base de dados de repasses do Ministério da Saúde, não são municípios e, portanto, não estão obrigados à realização de gasto mínimo em saúde nos termos da *Constituição Federal* de 1988 para a esfera municipal ^32^.

Os métodos para estimação dos valores transferidos pelo Ministério da Saúde por emendas parlamentares foram descritos por Vieira ^33^ e, para a despesa total dos municípios em APS, por Vieira et al. ^34^. Do gasto total em APS de cada município, deduziu-se o valor transferido pelo Ministério da Saúde para essa área, obtido dos arquivos de repasse do Fundo Nacional de Saúde (FNS), a fim de estimar a despesa própria do ente.

Os municípios foram agrupados em sete grupos, segundo o número de habitantes em 2015: 1 (até 5.000, 2 (de 5.001 a 10.000), 3 (de 10.001 a 20.000), 4 (de 20.001 a 50.000), 5 (de 50.001 a 100.000), 6 (de 100.001 a 500.000) e 7 (mais de 500.000) ^32^.

Como variáveis independentes, utilizaram-se os valores *per capita* alocados aos municípios brasileiros por meio de emendas parlamentares para a APS e de emendas parlamentares para incremento do PAB. Já as variáveis dependentes foram a despesa *per capita* em ações e serviços públicos de saúde com recursos próprios e a despesa municipal *per capita* com recursos próprios para APS (Quadro 1).

**Quadro 1:** Descrição das variáveis dependentes e independentes.

VARIÁVEL	NOME DO INDICADOR	DESCRIÇÃO	FORMA DE CÁLCULO	FONTE
Independente	Emendas parlamentares para APS	Despesa total liquidada pelo Ministério da Saúde em APS por emenda parlamentar, tendo o município como favorecido	Soma das despesas liquidadas em emenda parlamentar destinadas à APS (recursos de estruturação e custeio) nos anos de 2015 a 2020 *	Siga Brasil
	Emendas parlamentares para incremento do PAB	Despesa paga pelo Ministério da Saúde por emenda parlamentar para incremento do PAB, tendo como favorecido o município	Soma das despesas pagas pelo Ministério da Saúde para incremento do PAB (recursos de custeio) por emenda parlamentar nos anos de 2015 a 2020 *	Siga Brasil
Dependente	Despesa *per capita* em ações e serviços públicos de saúde com recursos próprios	Despesa do município em ações e serviços públicos de saúde com recursos próprios por habitante	Gasto informado pelo município em ações e serviços públicos de saúde com recursos próprios divididos por sua população no ano de referência **	SIOPS
	Despesa *per capita* em APS com recursos próprios	Despesa do município em APS com recursos próprios por habitante	Despesa liquidada do município em APS ***, subtraída do valor líquido repassado, do FNS para os Fundos Municipais de Saúde no custeio da APS, dividida pela população do município ^#^ nos anos de 2015 a 2020	SIOPS/FNS

APS: atenção primária à saúde; FNS: Fundo Nacional de Saúde; PAB: Piso da Atenção Básica; SIOPS: Sistema de Informações sobre Orçamentos Públicos em Saúde.

* Vieira ^33^;

** SIOPS (Ministério da Saúde ^56^);

*** Vieira et al. ^34^;

^#^ Arquivos de repasse do FNS (Ministério da Saúde ^57^) e Instituto Brasileiro de Geografia e Estatística ^58^^,^^59^.

### Análise dos dados

Os recursos financeiros (emendas e gastos) foram padronizados por meio da divisão pela população dos municípios, gerando valores *per capita* que foram corrigidos pela inflação oficial do período (Índice Nacional de Preços ao Consumidor Amplo - IPCA) para preços médios de 2020. As variáveis dependentes e independentes foram analisadas de forma descritiva, por meio da média e desvio padrão (DP), segundo o porte populacional dos municípios e o ano. Posteriormente, realizou-se o teste de Kolmogorov-Smirnov para a análise da normalidade e, para dados não normais, o teste de Kruskal-Wallis para identificar possíveis diferenças entre os grupos.

A análise da associação entre as variáveis independentes e dependentes se deu por meio de modelos de equações de estimativa generalizada (GEE) para medidas repetidas relacionadas, com estimativa do coeficiente beta (β) e intervalo de 95% de confiança (IC95%). Foram utilizados os seguintes parâmetros: distribuição normal da variável dependente, linear, matriz não estruturada e variância robusta ^35^^,^^36^^,^^37^^,^^38^. As análises foram realizadas de forma estratificada por porte do município e ajustadas, em modelo multivariado, para a obtenção do beta ajustado (β_aj_) ^35^^,^^36^^,^^37^, pelas variáveis: Unidade Federativa (UF); emendas; despesa liquidada *per capita* em APS; incremento do PAB; e um termo de interação entre as emendas (atenção básica liquidada e incremento do PAB). Além do cálculo empregado em todo o período de análise (2015 a 2020), foi realizado teste de sensibilidade com os dados referentes ao período de 2015 a 2019, ou seja, desconsiderando os dados do primeiro ano da pandemia de COVID-19. As análises foram realizadas com o suporte do programa SPSS, versão 25 (https://www.ibm.com/).

## Resultados

De acordo com a população estimada para 2015, os municípios estavam distribuídos conforme os portes: 1 (n = 1.236, 22,3%), 2 (n = 1.214, 21,8%), 3 (n = 1.374, 24,7%), 4 (n = 1.090, 19,6%), 5 (n = 351, 6,3%), 6 (n = 263, 4,7%) e 7 (n = 40, 0,7%). Do total de 5.568 municípios, 68,3% se enquadram nos grupos 1 a 3, com população inferior a 20 mil habitantes.

A Tabela 1 detalha os gastos com recursos próprios em ações e serviços públicos de saúde e APS, além dos repasses por emendas parlamentares (para APS e incremento do PAB *per capita*) recebidos pelos municípios brasileiros do período de 2015 a 2020, estratificados por porte populacional e ano.

No período avaliado, as despesas com recursos próprios em ações e serviços públicos de saúde não sofreram grandes variações, independentemente do porte municipal, tanto em percentual como em valores *per capita*, exceto o gasto em APS *per capita*, que apresentou tendência de queda no período (2015 a 2019), voltando a crescer em 2020.

**Tabela 1:** Despesas com saúde e emendas recebidas por municípios brasileiros, segundo ano e porte dos municípios (2015-2020).

Municípios (porte)	Total	2015	2016	2017	2018	2019	2020	Valor de p *
	Média (DP)	Média (DP)	Média (DP)	Média (DP)	Média (DP)	Média (DP)	Média (DP)	
Despesa com recursos próprios em saúde ** (em %)								
Total	22,10 (4,99)	22,00 (4,84)	21,64 (5,00)	22,82 (4,99)	21,69 (4,94)	21,67 (4,78)	22,78 (5,23)	< 0,001
1	20,75 (3,89)	20,52 (3,65)	20,12 (3,68)	21,48 (4,01)	20,35 (3,79)	20,45 (3,63)	21,57 (4,39)	< 0,001
2	21,68 (4,63)	21,50 (4,56)	21,17 (4,54)	22,40 (4,65)	21,38 (4,58)	21,37 (4,43)	22,36 (4,90)	< 0,001
3	22,45 (5,07)	22,29 (4,85)	21,92 (4,93)	23,21 (5,10)	21,98 (5,04)	22,03 (4,88)	23,27 (5,29)	< 0,001
4	22,61 (5,37)	22,71 (5,18)	22,14 (5,39)	23,37 (5,38)	22,13 (5,45)	21,98 (5,24)	23,17 (5,51)	< 0,001
5	23,52 (5,55)	23,61 (5,62)	23,48 (5,85)	24,19 (5,32)	23,09 (5,54)	22,94 (5,30)	23,82 (5,58)	0,028
6	24,13 (5,95)	24,18 (5,83)	24,34 (6,81)	24,62 (5,95)	23,67 (5,97)	23,62 (5,95)	24,54 (6,46)	0,221
7	25,01 (6,55)	25,05 (5,31)	25,71 (5,39)	25,39 (5,42)	24,31 (4,86)	23,90 (5,17)	25,11 (6,15)	0,568
Despesa com recursos próprios em saúde ** (em R$ *per capita*)								
Total	568,14 (364,15)	555,21 (355,88)	548,69 (348,61)	562,15 (352,96)	558, 87 (358,38)	587,65 (379,99)	596,40 (385,26)	< 0,001
1	947,86 (403,64)	912,46 (372,22)	905,72 (365,62)	935,85 (390,31)	935,76 (403,11)	991,38 (431,12)	1.007,17 (444,26)	< 0,001
2	542,78 (261,93)	530,62 (259,03)	529,01 (259,18)	543,08 (258,16)	539,89 (262,90)	567,65 (276,39)	576,10 (283,33)	< 0,001
3	441,75 (240,17)	436,44 (267,06)	429,05 (243,58)	438,39 (225,24)	431,16 (228,38)	454,54 (236,65)	458,17 (221,91)	< 0,001
4	398,94 (247,87)	396,88 (262,79)	385,52 (245,74)	393,76 (238,49)	389,42 (237,65)	403,86 (245,79)	411,85 (248,31)	0,031
5	402,50 (271,36)	404,48 (305,14)	397,31 (309,79)	401,35 (296,67)	401,55 (298,37)	413,85 (300,85)	419,47 (302,75)	0,775
6	476,79 (354,96)	473,41 (339,00)	461,79 (337,41)	460,00 (298,74)	462,89 (300,21)	482,39 (330,28)	486,16 (331,59)	0,751
7	511,92 (216,75)	516,62 (209,03)	514,91 (211,85)	515,77 (218,59)	505,23 (206,71)	517,09 (219,19)	527,91 (235,92)	1,000
Despesa com recursos próprios em APS (em R$ *per capita*)								
Total	437,84 (378,56)	450,31 (359,24)	445,18 (358,58)	429,75 (358,32)	415,64 (383,91)	413,94 (395,85)	472,62 (409,22)	< 0,001
1	836,66 (469,22)	831,59 (419,81)	825,76 (422,33)	810,20 (437,90)	820,55 (485,13)	834,62 (509,67)	906,55 (521,76)	0,001
2	444,90 (279,82)	455,41 (261,95)	458,07 (270,11)	435,09 (270,39)	420,57 (292,73)	421,29 (287,93)	494,64 (300,89)	< 0,001
3	320,03 (232,74)	339,71 (240,28)	331,67 (229,82)	316,62 (222,17)	296,69 (235,38)	288,91 (233,80)	346,54 (232,69)	< 0,001
4	254,97 (213,29)	277,23 (227,02)	266,25 (207,33)	256,48 (204,49)	226,34 (194,98)	217,79 (204,16)	266,81 (211,35)	< 0,001
5	224,58 (173,55)	246,29 (193,45)	236,66 (176,16)	231,29 (174,13)	212,14 (178,15)	198,19 (166,68)	224,91 (165,11)	< 0,001
6	219,19 (189,38)	239,40 (198,46)	239,21 (233,23)	226,67 (180,48)	201,80 (161,82)	192,39 (157,91)	208,29 (171,54)	0,005
7	190,43 (132,04)	206,78 (146,72)	199,04 (144,11)	195,94 (132,29)	190,12 (131,54)	181,92 (130,62)	188,61 (125,61)	0,986
Emendas para APS (em R$ *per capita*)								
Total	40,04 (55,87)	0,00 (0,03)	14,88 (29,40)	34,26 (47,96)	71,26 (67,81)	70,91 (58,66)	48,88 (56,99)	< 0,001
1	67,55 (77,06)	0,00 (0,00)	26,46 (45,29)	62,36 (63,31)	122,02 (84,11)	106,57 (72,01)	87,36 (78,33)	< 0,001
2	47,75 (56,23)	0,00 (0,03)	17,54 (28,78)	42,16 (49,78)	85,54 (61,18)	82,02 (56,39)	59,56 (54,06)	< 0,001
3	35,21 (44,58)	0,00 (0,00)	12,63 (21,45)	28,17 (37,09)	62,59 (51,79)	66,71 (49,62)	40,83 (39,33)	< 0,001
4	24,08 (34,84)	0,00 (0,03)	8,25 (16,19)	17,66 (29,05)	41,76 (41,09)	50,52 (41,66)	26,22 (30,99)	< 0,001
5	13,89 (23,44)	0,00 (0,02)	4,56 (8,69)	9,41 (18,43)	21,93 (26,55)	32,65 (30,75)	13,08 (19,69)	< 0,001
6	7,78 (14,61)	0,00 (0,07)	3,14 (6,85)	4,54 (10,29)	10,49 (15,01)	18,82 (20,31)	8,49 (14,70)	< 0,001
7	4,05 (9,14)	0,00 (0,00)	0,87 (1,34)	1,58 (3,96)	3,77 (9,26)	10,48 (13,57)	3,07 (5,12)	< 0,001
Emendas para incremento do PAB (em R$ *per capita*)								
Total	28,64 (45,93)	0,00 (0,02)	10,42 (25,08)	34,26 (47,96)	58,97 (59,64)	24,97 (34,22)	43,27 (51,66)	< 0,001
1	46,37 (63,48)	0,00 (0,00)	17,91 (37,69)	62,36 (63,31)	96,27 (72,94)	26,60 (40,52)	75,35 (70,64)	< 0,001
2	34,89 (48,34)	0,00 (0,03)	12,81 (25,67)	42,16 (49,78)	71,98 (58,23)	27,91 (36,37)	53,56 (50,09)	< 0,001
3	25,89 (37,47)	0,00 (0,00)	9,07 (19,47)	28,17 (37,09)	53,53 (47,99)	27,91 (33,38)	36,79 (36,89)	< 0,001
4	17,76 (29,59)	0,00 (0,00)	5,80 (14,99)	17,66 (29,05)	36,00 (40,41)	23,56 (29,98)	23,63 (29,71)	< 0,001
5	10,08 (19,57)	0,00 (0,02)	2,59 (7,71)	9,41 (18,43)	19,27 (26,27)	15,99 (22,86)	12,01 (19,21)	< 0,001
6	5,41 (11,88)	0,00 (0,07)	2,08 (6,08)	4,54 (10,29)	8,85 (14,92)	8,96 (14,05)	7,43 (13,56)	< 0,001
7	2,86 (7,52)	0,00 (0,00)	0,45 (1,22)	1,58 (3,96)	3,30 (9,09)	6,43 (11,18)	2,17 (3,58)	< 0,001

APS: atenção primária à saúde; DP: desvio padrão; PAB: Piso da Atenção Básica.

* Teste de Kruskal-Wallis;

** De acordo com a *Lei Complementar nº 141/2012*^60^.

Ao analisar as despesas com saúde, com foco no porte populacional (Tabela 1), observa-se que quanto maior o porte dos municípios, maior o percentual de recursos próprios alocados às ações e serviços públicos de saúde durante todo o período. No entanto, as despesas com recursos próprios em APS apresentaram um comportamento invertido, no qual municípios menores foram os que mais gastaram. Tiveram destaque aqueles com até 5 mil habitantes (porte 1), com gasto *per capita* médio em APS até 4,4 vezes superior ao dos municípios com mais de 500 mil habitantes (porte 7). Nos municípios do porte 1, o gasto *per capita* em APS, correspondeu a um valor bem próximo ao gasto *per capita* em ações e serviços públicos de saúde, diferentemente do que aconteceu para o grupo dos maiores municípios, cujo gasto *per capita* em APS correspondeu a menos da metade dos gastos com ações e serviços públicos de saúde.

As emendas para APS apresentaram tendência de aumento entre 2016 e 2018, com certa estabilidade em 2019 e redução em 2020, em todos os grupos de municípios. As emendas para incremento do PAB aumentaram aproximadamente 460% se comparado aos valores transferidos nos anos 2016 e 2018, período em que mantiveram uma expressiva tendência de alta, reduzindo abruptamente em 2019. Em 2020, os valores voltaram a aumentar, mas não atingiram o mesmo patamar de 2018.

Os valores *per capita* de emendas parlamentares, tanto para a APS quanto para incremento do PAB, aumentaram significativamente no período do estudo em todos os grupos, com maiores valores entre os anos de 2017 e 2019 (Tabela 1), e mostraram grande discrepância de valores entre os municípios de diferentes portes. Nas emendas para a APS, os municípios de porte 7 receberam, em média, um valor *per capita* até 16 vezes menor quando comparados aos de porte 1.

O recebimento de recursos federais por emendas parlamentares para a APS, que considera as emendas de investimento e custeio, associou-se à redução das despesas com recursos próprios municipais em APS (Tabelas 2 e 3), exceto para os municípios de porte 7 na análise de sensibilidade (dados de 2015-2019) (Tabela 3). Não se observou associação entre as emendas de incremento do PAB com a despesa com recursos próprios *per capita* em ações e serviços públicos de saúde e APS no período de 2015 a 2020.

O recebimento de emendas para APS associou-se ao aumento da despesa *per capita* com recursos próprios para municípios do porte 1 e 2 (Tabela 2) independentemente da pandemia de COVID-19 (Tabela 3). Para os demais grupos de municípios, essa associação não foi observada.

**Tabela 2:** Associação entre os recursos federais recebidos por emendas parlamentares e as despesas com recursos próprios em ações e serviços públicos de saúde e atenção primária à saúde (APS) dos municípios brasileiros, segundo porte populacional (2015-2020).

Municípios (porte)	Variáveis dependentes [β_aj_ * (IC95%; valor de p)]	
	Despesa com recursos próprios em ações e serviços públicos de saúde ** (em R$ *per capita*)	**Despesa com recursos próprios com APS (em R$ *per capita*)**
Emenda para APS (a cada R$ 10,00 *per capita*)		
1	6,59 (4,79; 8,39; < 0,001)	-5,85 (-7,39; -4,29; < 0,001)
2	3,19 (1,37; 5,00; < 0,001)	-10,29 (-11,66; -8,91; < 0,001)
3	1,19 (-0,64; 3,02; 0,201)	-7,19 (-8,34; -6,03; < 0,001)
4	0,43 (-1,26; 2,12; 0,617)	-11,35 (-13,12; -9,59; < 0,001)
5	-0,53 (-2,27; 1,25; 0,559)	-14,77 (-19,99; -9,54; < 0,001)
6	4,50 (-3,07; 12,08; 0,244)	-18,27 (-31,05; -5,49; 0,005)
7	-18,91 (-46,57; 8,75; 0,180)	-8,74 (-15,87; -1,61; 0,016)
Emendas para incremento do PAB (a cada R$ 10,00 *per capita*)		
1	1,72 (-2,74; 6,18; 0,449)	-1,04 (-3,86; 1,77; 0,467)
2	-0,24 (-2,77; 2,29; 0,851)	0,77 (-1,37; 2,91; 0,480)
3	-0,89 (-3,69; 1,92; 0,236)	-1,63 (-3,26; 0,00; 0,050)
4	-2,72 (-5,95; 0,51; 0,099)	0,50 (-2,31; 3,31; 0,726)
5	2,49 (-1,28; 6,27; 0,195)	-1,38 (-6,51; 3,74; 0,597)
6	-2,17 (-13,02; 8,69; 0,696)	-2,70 (-25,86; 20,45; 0,819)
7	28,88 (-1,77; 59,31; 0,138)	-9,28 (-37,84; 19,28; 0,524)

IC95%: intervalo de 95% de confiança; PAB: Piso da Atenção Básica.

* Coeficiente beta ajustado por Unidade Federativa por emendas para atenção básica liquidadas, emendas para incremento do PAB e interação entre emendas para atenção básica liquidadas e emendas para incremento do PAB;

** De acordo com a *Lei Complementar nº 141/2012*^60^.

**Tabela 3:** Associação entre os recursos federais recebidos por emendas parlamentares e as despesas com recursos próprios em ações e serviços públicos de saúde e atenção primária à saúde (APS) dos municípios brasileiros, segundo porte populacional, excetuando o ano de 2020 em razão da pandemia de COVID-19 (2015-2019).

Municípios (porte)	Variáveis dependentes [β_aj_ * (IC95%; valor de p)]	
	Despesa com recursos próprios em ações e serviços públicos de saúde ** (em R$ *per capita*)	**Despesa com recursos próprios com APS (em R$ *per capita*)**
Emenda para atenção básica liquidada (a cada R$ 10,00 *per capita*)		
1	4,14 (3,17; 5,09; < 0,001)	-3,40 (-4,54; -2,05; < 0,001)
2	1,77 (0,87; 2,66; < 0,001)	-4,58 (-5,67; -3,49; < 0,001)
3	1,40 (-0,52; 3,32; 0,153)	-2,93 (-4, 14; -1,45; < 0,001)
4	0,73 (-1,16; 3,08; 0,540)	-8,34 (-11,28; -5,39; < 0,001)
5	0,65 (-1,44; 2,73; 0,545)	-15,33 (-22,31; -8,35; < 0,001)
6	-6,51 (-17,64; 4,61; 0,251)	-19,65 (-31,35; -7,94; 0,001)
7	-3,88 (-22,76; 14,99; 0,687)	-10,14 (-21,31; 1,03; 0,075)
Emendas para incremento do PAB (a cada R$ 10,00 *per capita*)		
1	-1,03 (-3,36; 1,29; 0,386)	-1,95 (-4,54; 0,64; 0,140)
2	-4,95 (-6,86; -3,05; < 0,001)	-2,57 (-4,46; -0,68; 0,008)
3	-1,35 (-4,45; 1,76; 0,396)	-6,81 (-9,64; -3,98; < 0,001)
4	-6,44 (-10,09; -2,78; 0,001)	-3,69 (-7,25; -0,15; 0,041)
5	1,82 (-1,54; 5,18; 0,289)	-1,41 (-7,92; 5,11; 0,672)
6	10,60 (-10,07; 31,27; 0,315)	-2,11 (-26,48; 22,26; 0,865)
7	21,36 (-18,03; 60,75; 0,288)	-3,81 (-25,35; 17,73; 0,729)

IC95%: intervalo de 95% de confiança; PAB: Piso da Atenção Básica.

* Coeficiente beta ajustado por Unidade Federativa por emendas para atenção básica liquidadas, emendas para incremento do PAB e interação entre emendas para atenção básica liquidadas e emendas para incremento do PAB;

** De acordo com a *Lei Complementar nº 141/2012*^60^.

## Discussão

São três os principais achados deste estudo para a análise do significado e das consequências da ampliação da participação do repasse federal de emendas parlamentares para o financiamento municipal do SUS e, particularmente, da APS.

O primeiro é a existência de um grande desequilíbrio na alocação de recursos do Ministério da Saúde por emendas parlamentares, segundo o porte populacional dos municípios, em favor dos que têm menor população. Foram identificadas diferenças de até 16 vezes no valor *per capita* em emendas parlamentares para APS entre os municípios com até 5 mil habitantes e aqueles com mais de 500 mil, evidenciando as distorções causadas por essa forma de transferência de recursos no período avaliado. O fato é que as desigualdades encontradas na alocação de recursos para o financiamento de ações e serviços públicos de saúde por emendas parlamentares precisam ser investigadas em detalhes, pois, em vez de corrigir as iniquidades na distribuição dos recursos, essa modalidade de alocação pode aumentá-las.

Essa situação já havia sido apontada em estudo que considerou a alocação federal para incremento do PAB no período de 2015 a 2018 ^10^. Outros dois trabalhos indicam distorções causadas pelas emendas parlamentares, perante os mecanismos de transferência federal para a atenção básica previamente instituídos no SUS ^26^ e a concentração de recursos em municípios que precisavam menos do repasse ^24^.

Nesse sentido, em relação à grande vulnerabilidade dos municípios de menor porte populacional na Federação ^39^ e à sua alta dependência fiscal das transferências federais ^40^, sabe-se que o repasse de emendas parlamentares não beneficia a todos de forma equitativa, dada a discricionariedade do parlamentar na indicação desses recursos ^26^. Mesmo considerando a insuficiência dos valores praticados, o PAB fixo que vigorou até 2019 favoreceu o fortalecimento da APS desde a sua implantação nos anos 1990, na medida em que respeitou critérios como perfil demográfico e socioeconômico de cada ente municipal para a distribuição de recursos financeiros ^41^.

Outro importante ponto que repercute sobre os valores *per capita* de distribuição das emendas parlamentares diz respeito à divisão dos recursos no Congresso Nacional, tendo em vista que os valores são determinados pelo número de parlamentares de cada UF e não pela população em si. Ao considerar os estados de população pequena em relação ao número de cadeiras no Congresso Nacional, pode-se dizer que esses locais estavam sobrerrepresentados e favorecidos com maior proporção de recursos federais ^42^. Esse assunto é tema de impasses entre os parlamentares pelo expressivo montante de recursos financeiros envolvidos. As distorções das emendas individuais e de relator são tantas que os municípios poderiam ser contemplados com receitas que extrapolam seu próprio orçamento anual ^43^.

Preocupações sobre os efeitos da distribuição de recursos federais por emendas parlamentares datam de um período em que a execução das despesas por esse mecanismo se mantinha em patamar baixo ^20^. Com a ampliação dos recursos destinados por emendas de relator, com baixa transparência sobre a finalidade da alocação pelo parlamentar que destina recursos e sobre os entes beneficiados, essas preocupações aumentaram consideravelmente ^10^^,^^34^^,^^44^. Isso porque os recursos de emendas parlamentares alocados pelo Ministério da Saúde são contabilizados para efeito da vinculação constitucional das despesas do ente federal em ações e serviços públicos de saúde. Na vigência do teto de gastos, a relevância de critérios políticos na alocação de recursos sugere maior fragmentação e dificuldades para suprir as necessidades de financiamento do SUS, de forma coerente e articulada ^13^.

O segundo achado do estudo é a inexistência de correlação entre a alocação de recursos federais por emendas parlamentares aos municípios para financiamento da APS e a despesa com recursos próprios em ações e serviços públicos de saúde, exceto para aqueles com população inferior ou igual a 10 mil habitantes, para os quais a associação foi positiva. Esses achados apresentam evidências que refutam a hipótese da substituição de fontes pelos municípios no financiamento de despesas em ações e serviços públicos de saúde, ou seja, de que eles substituiriam recursos próprios por emendas na aplicação em ações e serviços públicos de saúde.

Como a aplicação municipal efetiva em ações e serviços públicos de saúde, em média, foi de 22% no período analisado, sendo muito superior ao limite mínimo de 15% estabelecido na *Constituição Federal*, os municípios teriam margem para realizar essa substituição sem descumprir a determinação constitucional. Contudo, isso não aconteceu. A pressão sobre os municípios, decorrente do subfinanciamento do SUS, é um dos fatores que explicam esse resultado ^45^.

A disparidade entre os entes da Federação no financiamento do SUS reflete a omissão estrutural da União quanto ao seu papel coordenador e organizativo dentro do arranjo federativo ^1^. A participação da União, que nos anos 1990 correspondia a aproximadamente 73% do financiamento da saúde, reduziu para 42% em 2019. Em contrapartida, a alocação de recursos da esfera municipal, nessa mesma área, passou de 12% para cerca de 31% no mesmo período ^27^^,^^46^. Tal fato indica um paradoxo nesse cenário de inversão de responsabilidades, uma vez que a União tem a maior concentração tributária e, por consequência, a maior arrecadação de receitas ^1^.

Essa situação também evidencia que a descentralização da gestão das ações e serviços públicos de saúde aos municípios não teve como prioridade a garantia do acesso universal aos serviços de saúde, mas sim um caráter estratégico de intervenção na economia, com o projeto de enxugamento do Estado e de estabilidade econômica ^47^. Ou seja, representou mais uma estratégia de deslocamento da responsabilidade sobre o gasto social para as esferas subnacionais, servindo mais ao propósito de retração da União e de contenção de despesas do que de sua expansão, como almejavam seus idealizadores ^48^.

Entre as despesas que recaem e têm grande impacto no orçamento municipal da saúde, estão as de natureza remuneratória, sejam elas decorrentes dos cargos em exercício, como servidores e empregados públicos ou funções de confiança, ou dos diversos tipos de obrigações trabalhistas de responsabilidade do empregador ^49^. Esse é um dos maiores desafios encontrados pelos gestores municipais: garantir o provimento da força de trabalho de maneira suficiente dentro da limitação imposta pela *Lei de Responsabilidade Fiscal*^50^.

Por outro lado, as emendas parlamentares individuais de custeio, que têm representado relevância no financiamento da APS para muitos municípios e na maior parte são repassadas como incremento ao PAB, têm sua destinação vedada para o pagamento de pessoal ou encargos sociais ^51^. Com isso, é possível que parte desses recursos tenham se destinado às contratações por meio de pessoa jurídica, direcionando essas receitas para as despesas com serviços de terceiros. Estudo realizado no Estado do Paraná corrobora essa hipótese ao revelar que, entre os anos de 2015 e 2020, houve aumento expressivo (superior a 500%) do número de trabalhadores vinculados ao SUS na modalidade de pessoa jurídica, fenômeno que ficou conhecido como “*pejotização*” ^52^. No entanto, são necessárias pesquisas que aprofundem a análise da destinação dos recursos recebidos por emendas parlamentares pelos municípios, para afirmar que, em alguma medida, as emendas parlamentares tenham contribuído para a precarização das relações de trabalho.

O terceiro achado está relacionado à identificação de associação entre a alocação de recursos federais por emendas parlamentares para a APS e a redução da despesa com recursos próprios em APS, o que sugere um efeito de substituição nessa destinação específica. Ou seja, os municípios diminuíram a parcela de recursos próprios no financiamento da APS. Mas, como não houve redução da despesa própria em ações e serviços públicos de saúde, o montante de recurso próprio que deixou de ser aplicado em APS foi deslocado para o financiamento de outras despesas, mesmo em municípios de pequeno porte, nos quais a oferta de serviços se concentra nesse nível de atenção à saúde. Para esses entes, e mesmo em municípios maiores, os recursos podem ter sido destinados ao financiamento de ações de vigilância em saúde, para a aquisição de medicamentos, ou mesmo ao financiamento de serviços de saúde de média e alta complexidade ^34^, uma vez que esses itens de gasto não estão incluídos na despesa em APS considerada nesta análise.

Ressalta-se que, mesmo antes da vigência do orçamento impositivo, a APS já constava entre as áreas prioritárias de financiamento do Ministério da Saúde. Mas foi a partir de 2016 que passou a figurar como a principal linha de alocação de recursos por emendas parlamentares aos municípios. Tal situação pode estar relacionada à maior facilidade de transferência de recursos para essa ação específica. Enquanto a totalidade de municípios brasileiros pode receber emendas parlamentares no componente da APS, tanto na modalidade manutenção como na modalidade estruturação ^10^, os recursos para média e alta complexidade estão limitados aos entes e instituições filantrópicas que executam esses serviços ^53^. Essa é uma realidade diferente dos municípios de pequeno porte populacional, principais beneficiários das transferências por emendas parlamentares ^26^.

Dessa forma, além da necessidade de se investigar para quais finalidades os recursos municipais para a APS foram realocados, importa saber se esses recursos foram utilizados de maneira mais efetiva pelos governos locais. Diversos estudos indicam as dificuldades e os desafios para que os serviços de atenção básica se constituam como porta de entrada e centro de comunicação das complexas redes de cuidado no SUS ^54^. A estabilidade e a suficiência de recursos para seu financiamento são condições essenciais para melhorar o acesso, a equidade e a qualidade da APS no SUS.

Quanto às limitações do estudo, uma está relacionada aos dados disponibilizados no Sistema de Informações sobre Orçamento Público em Saúde (SIOPS), pelo fato de as informações lá contidas serem autoinformadas. Outra está na estimação do gasto próprio dos municípios em APS, utilizada como variável dependente, em que não foi possível deduzir a parcela de transferência do estado para esses entes, por dificuldades metodológicas envolvendo a disponibilidade de dados no SIOPS. Contudo, é provável que o impacto dessa situação sobre os resultados seja pequeno, na medida em que a participação dos estados no financiamento da APS é baixa. Na média do período de 2015 a 2019, a participação dos estados foi de 7,4% da atenção curativa ambulatorial básica ^55^ e parte dos recursos alocados é executada diretamente pelos estados e não na modalidade de transferência aos municípios. Em relação ao modelo estatístico adotado, consideram-se como limitações, ainda, a falta de uma variável de ajuste invariante ao longo do tempo (UF), a ausência de um modelo teórico que pudesse direcionar a inclusão de mais variáveis de ajustes às análises, e a eventual indisponibilidade desses dados.

Tais limitações não comprometem a relevância deste trabalho, cujos resultados confirmam que, após a aprovação das regras de impositividade para a execução orçamentária, houve um aumento expressivo dos repasses por emendas parlamentares sobre o montante de recursos financeiros transferidos da União para os municípios ^27^^,^^33^^,^^34^. A alocação desses recursos reflete: desequilíbrios e efeitos diferenciados sobre os gastos municipais em ações e serviços públicos de saúde e APS, considerando os portes populacionais dos municípios, a substituição de fontes para o financiamento da APS, e o redirecionamento de recursos próprios para outras prioridades de gasto definidas pela gestão local.
